# Psychosocial determinants of sexual health in newly married couples: a protocol for a mixed-methods study

**DOI:** 10.1186/s12978-023-01705-w

**Published:** 2023-10-24

**Authors:** Zahra Toghiani, Ashraf Kazemi, Mahbobeh Taebi

**Affiliations:** 1 Student Research Committee, School of Nursing and Midwifery, Isfahan University of Medical Sciences, Isfahan, Iran; 2 Reproductive Health Department, School of Nursing and Midwifery, Isfahan University of Medical Sciences, Hezarjerib AV., Isfahan, Iran; 3 Midwifery and Reproductive Health Department, School of Nursing and Midwifery, Isfahan University of Medical Sciences, Isfahan, Iran

**Keywords:** Psychosocial determinants, Sexual health, Couples

## Abstract

**Background:**

Regarding psychosocial factors affecting sexual health and in most cultures, healthy and pleasurable sexual relations are valued in the family context, the present study aims to identify the psychosocial determinants of sexual health in newly married couples.

**Methods:**

The present mixed-methods study will be conducted in three phases. The first phase will be carried out qualitatively based on which psychosocial determinants of the sexual health of newly married couples will be extracted. In the second phase, questionnaire items of psychosocial determinants of sexual health in newly married couples will be compiled, and face validity (quantitative and qualitative), content validity (quantitative and qualitative), and reliability of the questionnaire will be evaluated. In the third phase, which will be the quantitative phase of the study, the construct validity of the questionnaire will be assessed by analyzing the main items. Moreover, at this stage, the relationship between the extracted psychosocial determinants and the sexual health of newly married couples will be investigated.

**Discussion:**

By explaining the psychosocial determinants of sexual health in newly married couples, it is possible to identify key variables for designing interventions that improve the sexual health of this group, based on their cultural sensitivities.

## Background

Health is one of the criteria for the development of countries [1]. Sexual health as a part of individual and social health is a need and strategy to achieve the millennium development goals [2], which has diverse and complex dimensions and impacts marital satisfaction and stability [3]. A national survey in Iran has shown that 56% of couples applying for divorce were dissatisfied with their sex life [4]. In addition, lack of sexual satisfaction among couples is one of the causes of family breakups [5], which leads to high-risk sexual behaviors [6, 7], behaviors that directly jeopardize the couples’ sexual health [8–10].

The results of studies show that lack of sexual satisfaction [11] and high-risk sexual behaviors [12] are associated with various domestic violence and threaten the mental health of men and women [12–14]. Furthermore, each issue can increase the likelihood of extramarital relations or be affected by it [15–17]. The increased probability of sexually transmitted diseases [18], unwanted pregnancy, abortion, family instability [19], and increased psychosocial pressure on individuals [20] are some of the undesired consequences of extramarital relations.

Although studies in the field of sexual health are extensive, in most research, sexual health has been investigated through a biomedical approach, which focuses on identifying sexual disorders and examines sexual health as an individual and couple problem. This is while, according to the definition of world health organization, human sexuality is formed through the interactions between the individual and the society.

Therefore, it is necessary to consider sexual health as a social issue [21]. As a result, improving sexual health requires a sociological and psychological understanding of sexual health to consider individuals in their psychological, social, cultural, geographical, and political contexts [22, 23]. In order to prevent these psychological and social complications, planning for a satisfying and violence-free sexual life for couples is a key strategy to ensure sexual health and fertility.

Regarding psychosocial factors affecting sexual health, the biopsychosocial paradigm is raised. Based on this paradigm, barriers to sexual health might be physical, psychological, and social [24]; therefore, in order to provide sexual health promotion programs, in addition to a broad focus on understanding the physical causes of sexual problems and behaviors, it is necessary to investigate the interaction between physical, psychological, and social aspects related to sexual health and well-being [25, 26].

In this paradigm, psychological determinants, including cognitions, emotions, and personality traits, are investigated along with social determinants as factors influencing sexual performance and behavior [24]. The results of various studies have shown that sexual behavior and attitudes, social and cultural factors [27–29], biological risk and genetic predisposition, and physical [30, 31] and mental [32] diseases affect sexual health.

Although these factors have been proposed as significant determinants in the creation of sexual health in different societies, the way these factors interact and impact sexual health is formed in the social context. In Iran, the communication complexities and the expansion of social networks and virtual space, along with traditional norms and values, have altered individuals’ attitudes, values, expectations, and lifestyles of adolescents [33, 34]. These changes are distinct in men and women since men in these societies enjoy more courage and freedom to satisfy their sexual needs, which may cause contradictions in their married life, sexual demands, and expectations.

Therefore, effectual planning to improve the sexual health of new couples requires the identification of the psychosocial determinants of sexual health in this relatively emerging social context. Since, in most cultures, healthy and pleasurable sexual relations are valued in the family context, the aim of the present study, designed as a mixed-method study in 3 phases, is to identify the psychosocial determinants of sexual health in newly married couples.

## Methods

This exploratory sequential mixed-method study will be conducted in Isfahan, Iran in 3 phases with the approval of the Ethics Committee of Isfahan University of Medical Sciences (IR.MUI.NUREMA.REC.1401.130). The first phase will be carried out qualitatively based on which psychosocial determinants of the sexual health of newly married couples will be extracted. In the second phase, questionnaire items of psychosocial determinants of sexual health in newly married couples will be compiled, and face validity (quantitative and qualitative), content validity (quantitative and qualitative), and reliability of the questionnaire will be evaluated. In the third phase, which will be the quantitative phase of the study, the construct validity of the questionnaire will be assessed by analyzing the main items. Moreover, at this stage, the relationship between the extracted psychosocial determinants and the sexual health of newly married couples will be investigated.

### First phase: qualitative phase of the study

The purpose of the qualitative study is to explain the psychosocial determinants of the sexual health of newly married couples, which will be conducted through the qualitative content analysis approach.

In this phase of the study, the participants will include couples intending to get married, couples who have started living together, and those that have been married for a maximum of 5 years. Furthermore, at this stage of the study, family counseling, judiciary, and sexology experts with a history of providing counseling to couples will be included in the study. The inclusion criteria for the participants will include heterosexuality, ability to understand the questions, no known physical and mental disorders based on the medical history, and no behavioral disorders. Inclusion criteria for expert participants will include at least 5 years of work experience.

The participants will be selected from premarital counseling centers, sex counseling centers, comprehensive health service centers, and family courts of Isfahan using the purposeful sampling method. After identifying eligible participants and explaining the study objectives and participation, they will be invited to participate in the study.

It will be explained to them that participating and responding to the questions will be voluntary, and accepting or refusing them will not affect the services provided to them. In addition, they will be provided with explanations about the confidentiality of the information and the possibility of withdrawing from the study at each stage.

After obtaining informed consent, the interview time and location will be determined, and the interviews will be implemented with the coordination and opinion of the participants near their residences or in a private room in the mentioned centers. The fare will be borne by the researchers. Interviews with experts will also be conducted, if desired, at the workplace clinic or consulting offices, court, in a place close to their residence, or through software such as Skype.

The main data collection method will be semi-structured in-depth individual and paired interviews using guide questions. In order to compile the guide questions, 5 unstructured interviews will be conducted with the eligible candidates. The results of these interviews will not be included in the final analysis.

To deepen the interviews and obtain richer data, an exploratory question, such as “Can you explain this through more examples?” will be used. The interviews will be conducted by a female reproductive health Ph.D. student under the supervision of a female reproductive health specialist. A number of preliminary interviews will be conducted in the presence of the research officer, and the communication technique with the participant and raising the questions will be evaluated by her and modified if necessary.

Upon their request, individual interviews with men will be conducted by a male interviewer, a PhD student in counseling. Interviews will continue until data saturation. At the beginning of the interview and before its formal start, a short oral explanation about the study objectives will be presented to the participants. In addition to the prior coordination, the researcher will introduce herself once more, explain the continuation of the collaboration, and conduct subsequent interviews, if necessary, and permission to record the interview will be obtained.

The formal interview will be conducted by recording audio using a digital recording device. According to the participants’ conditions, the interview may be postponed or stopped and resumed on another day. The recorded interviews will only be available to the research team and will be deleted after implementation. Moreover, field notes will be used to collect data; therefore, in order to take field notes, the researcher will pay attention to the non-verbal behaviors, attitudes, and interactions of the participants before, during, or at the end of the interviews and will write them down.

Data will be analyzed simultaneously with data collection using the conventional content analysis method, according to Graneheim and Lundman [35]. The data analysis phase will begin when the researcher records or transcribes the interviews. The initial coding will start simultaneously with the interview, and the meaning units will be summarized, checked, and labeled with the main codes.

To evaluate the validity and reliability of the data, the criteria of credibility, dependability, confirmability, and transferability, will be used to measure scientific accuracy. For credibility, the member check technique will be used to confirm the reliability of the data and codes. To establish dependability, primary codes and samples of quotations, topics, and items extracted from the interview transcripts for each category will be provided to the external observer.

To ensure confirmability, the transcripts of several interviews and the extracted codes and categories will be provided to the research team, three reproductive health, and psychologists, and they will be requested to evaluate the accuracy of the data coding process. In order to establish the transferability of the findings, the participants’ statements will be presented as objectively as possible.

### Second phase: designing a questionnaire of psychosocial determinants of sexual health

In the second phase of the study, based on the findings from the first phase and the information obtained from the literature review, the items of the tool for the determinants of sexual health in newly married couples will be compiled. The method proposed by Creswell and Clark will be used in the design of the questionnaire for psychosocial determinants of sexual health in newly married couples [36]. Afterward, the face and content validity (quantitative and qualitative) of the designed questionnaire will be evaluated.

In order to evaluate the psychometric properties of the designed questionnaire, face and content validity will be determined quantitatively and qualitatively. For the qualitative face validity, the questionnaire will be provided to ten reproductive health and family counseling specialists and psychologists, and they will be requested to provide their modifying opinions about the items. For quantitative face validity, the item impact method will be used to reduce and eliminate disproportionate items and determine the significance of each item.

For the qualitative content validity, 20 experts (reproductive health, psychologists, and consultants) will be requested to share their written opinions regarding the content, Persian grammar, wording appropriateness, necessity, importance, and the proper placement of phrases. After collecting opinions and during a meeting with the research team, the proposed opinions will be reviewed, and appropriate modifications will be applied. For the quantitative content validity of the tool, the methods of calculating the content validity ratio (CVR) [37] and the content validity index (CVI) [38] will be used.

### Third phase: a cross-sectional study

The third phase of the study will be conducted as cross-sectional in two stages. In this phase, during the first stage, the construct validity of the psychosocial determinants of newly married couples’ sexual health questionnaire will be evaluated using main factor analysis. The tool’s reliability will also be assessed using the internal consistency evaluation method (calculation of Cronbach’s alpha, considering the coefficient of 0.7 (as the lower limit) and test–retest reliability before and after factor analysis with 10% of the samples. Afterward, in the second step, the relationship between the psychosocial determinants and the sexual health of newly married couples will be determined, and the fit of the extracted conceptual model will be evaluated.

The study setting will be comprehensive health service centers in Isfahan, and the participants will include newly married couples who refer to these centers. The inclusion criteria will include willingness to participate in the study and informed consent to share information, couples with Iranian nationality who have lived together for a maximum of 5 years, the ability to understand the questions or the lowest literacy to read and write, no known mental disorders or physical diseases based on health record, not being pregnant, and no history of infertility (in couples).

In this phase, during the first stage, the sample size will be determined to decide on the tool’s factor structure based on the sample size required for factor analysis. Five items will be considered for each item. Afterward, during the second stage and after extracting the factors, the study will be resumed with 384 couples, calculated based on the Cochran sample size formula for cross-sectional studies.

The sampling will be implemented through the cluster method. The 1 and 2 health systems of Isfahan will be considered as a category, and comprehensive health service centers covered by them as a cluster. Five centers will be randomly selected from each cluster using a table of random numbers. Afterward, depending on the required number of samples, n couples meeting the criteria will be selected from each center to enter the study.

The main method of data collection will be completing a self-report questionnaire. The first phase will include completing questionnaires related to the evaluation of demographic information and psychosocial determinants of the sexual health of newly married couples. The second phase will be completing questionnaires related to the evaluation of demographic information, couples’ marital satisfaction, and psychological determinants.

After obtaining permission from Isfahan University of Medical Sciences, the researcher will refer to selected comprehensive health service centers and will invite newly married couples eligible to participate in the study. To this end, the study objectives will be explained to them, and in case of their willingness to participate in the study, written, informed consent will be obtained. Due to the possibility of couples’ failure to refer to comprehensive health service centers simultaneously, the necessary explanations will be provided to the referring individual, and if he/she agrees to participate in the study, their spouse will also be invited to participate via phone.

### Data collection tools

The data collection tool will include the questionnaire for record the demographic information. This questionnaire includes questions related to personal and social characteristics (9 questions), including age, level of education, employment status, residence status, personal income, duration of marriage, type of marriage, and ethnicity. Also, marital satisfaction and psychosocial determinants of sexual health will be assessed, using validated self-reported questionnaires.

For assessing couples’ marital satisfaction, the ENRICH Marital Satisfaction questionnaire will be used. The original version of the questionnaire includes 125 questions in 14 subscales. In this study, subscales of marital satisfaction (10 questions), communication (10 questions), and sexual relation (10 questions) will be evaluated. Questions was scored on a 5-point Likert scale from ‘strongly disagree’ to ‘strongly agree,’ coded from 1 to 5, respectively. A higher score indicates greater satisfaction with married life [39].

#### Psychosocial determinants of sexual health

Questionnaire for assessing psychosocial determinants of sexual health of newly married couples.

Windows SPSS and AMOS software version 21 will be used for data analysis. Frequency distribution tables, graphs, mean, and standard deviation indices will be used to describe the characteristics of the studied subjects and determine questionnaire information. In order to evaluate the sampling adequacy and data appropriateness, Kaiser-Meyer-Elkin (KMO) and Bartlett’s test will be used. In order to extract the factors, the maximum likelihood factor analysis method will be used, and to obtain independent factors, Varimax orthogonal rotation will be employed. First-order confirmatory factor analysis will be performed using the maximum likelihood method, and goodness of fit indices will be evaluated. The Root Mean Square Error of Approximation (RMSEA) will be used and be considered acceptable if it is less than 0.08. Moreover, the Minimum Discrepancy Function divided by Degrees of Freedom (CMIN/DF) will be calculated, and if it is less than 3 and P > 0.05, the fit of the model will be considered appropriate. The correlation of variables will be evaluated using Pearson or Spearman correlation coefficient and multivariate linear regression and accepting a maximum error of 0.05.

## Discussion

This study will be conducted as a mixed method to determine the psychosocial determinants of the sexual health of newly married couples. Considering that sexual health is part of individuals’ and couples’ reproductive rights, it is necessary to clarify the facts about sexual health. The significant point in dealing with sexual health is to investigate it through a biological and psychosocial approach.

In addition to the biological factors affecting sexual health, it is also necessary to identify influencing psychosocial factors since dealing with sexual health without considering its psychosocial dimensions will result in the failure of sexual health promotion interventions.

Identifying the psychosocial dimensions of the sexual health of couples in a society requires conducting research in the specific cultural context of that society. Qualitative research is one of the best research types in these cases since, through qualitative research, it is possible to obtain a more profound view and more objective data about the individuals’ unique experiences [40]. Therefore, due to the ambiguity of the psychosocial determinants of the sexual health of newly married couples in Iranian society, the first phase of this study will be conducted qualitatively to identify the psychosocial determinants of the sexual health of newly married couples. On the other hand, due to the lack of reliable tools to evaluate these determinants in newly married couples in domestic and foreign studies, the second phase will be conducted in order to design a questionnaire of psychosocial determinants of the sexual health of newly married couples based on the results of the first phase and review of related literature.

In the third phase of the study, after evaluating the construct validity of the above instrument through a cross-sectional study, the relationship between the psychosocial determinants of sexual health and the marital satisfaction of newly married couples will be quantitatively evaluated; as a result, in addition to assessing the fit of the extracted conceptual model, key variables of designing an intervention program to improve the health of newly married couples are identified. The results of the third phase will be used in the fourth phase to design strategies to improve the sexual health of newly married couples.

Designing these approaches based on the identified key variables increases their effectiveness in the target group. After evaluating the applicability of the compiled strategies in a pilot study, they will be presented to health policymakers to improve the sexual health of newly married couples and strengthen the family foundation. In addition, these approaches can be used in other traditional societies with a culture similar to the Iranian community to improve the sexual health of couples.

## Conclusion

By explaining the psychosocial determinants of sexual health in newly married couples and designing a valid questionnaire to measure it in Iranian society, it is possible to have a tool that can be used to measure the psychosocial determinants of sexual health in newly married couples, which helps identify key variables for designing interventions that improve the sexual health of this group, based on their cultural sensitivities.

## Data Availability

Data and material will be available on request from the corresponding author.
